# Developing an interpretable machine learning predictive model of chronic obstructive pulmonary disease by serum PFAS concentration

**DOI:** 10.3389/fpubh.2025.1602566

**Published:** 2025-07-10

**Authors:** Xiaomei Shao, Ling Zhang, Yuting Wang, Youmei Ying, Xueqin Chen

**Affiliations:** ^1^Nanjing Jiangbei Hospital, Affiliated Nanjing Jiangbei Hospital of Xinglin College, Nantong University, Jiangsu, China; ^2^Huai'an No. 3 People's Hospital, Huaian Second Clinical College of Xuzhou Medical University, Jiangsu, China; ^3^The Affiliated Taizhou People's Hospital of Nanjing Medical University, Taizhou School of Clinical Medicine, Nanjing Medical University, Taizhou, Jiangsu, China

**Keywords:** chronic obstructive pulmonary disease, machine learning, partial dependence plot, SHapley additive exPlanations, environment pollution

## Abstract

**Background:**

Chronic obstructive pulmonary disease (COPD) is a leading cause of morbidity and mortality worldwide, with limited early detection strategies. While previous studies have examined the relationship between per- and polyfluoroalkyl substances (PFAS) and COPD, limited research has applied interpretable machine learning (ML) techniques to this association.

**Methods:**

We investigated the association between PFAS exposure and COPD risk in 4,450 National Health and Nutrition Examination Survey (NHANES) participants from 2013 to 2018. After excluding missing covariates and extreme PFAS values and applying K-nearest neighbors (KNN) imputation, nine ML models, including CatBoost, were built and evaluated using metrics like accuracy, area under the curve (AUC), sensitivity, and specificity. The best-performing model was further analyzed using partial dependence plots (PDP) and SHapley additive exPlanations (SHAP) analysis. To enhance clinical applicability, the final model was deployed as a publicly accessible web-based risk calculator.

**Results:**

CatBoost emerged as the best model, achieving an accuracy of 84%, AUC of 0.89, sensitivity of 81%, and specificity of 84%. PDP revealed that higher perfluorooctane sulfonic acid (PFOS) and perfluoroundecanoic acid (PFUA) levels were associated with reduced COPD risk, whereas perfluorooctanoic acid (PFOA) and 2-(N-Methyl-perfluorooctane sulfonamido) acetic acid (MPAH) showed positive associations with COPD. perfluorononanoic acid (PFNA), perfluorodecanoic acid (PFDE), and perfluorohexane sulfonic acid (PFHxS) demonstrated mixed or non-linear effects. SHAP analysis provided insights into individual predictions and overall variable contributions, clarifying the complex PFAS-COPD relationship. The deployed web-based calculator enables interactive prediction and risk interpretation, supporting potential public health applications.

**Conclusion:**

CatBoost identified PFOS and PFUA as protective factors against COPD, while PFOA and MPAH increased risk of COPD. These findings emphasize the need for stricter PFAS regulation and highlight the potential of machine learning in guiding prevention strategies.

## Introduction

### Global burden and trends of COPD

Chronic obstructive pulmonary disease (COPD) is a major global health issue, affecting an estimated 328 million people worldwide (1–3). While smoking is the leading cause, other factors such as biomass fuel exposure, occupational hazards, and air pollution also contribute significantly, especially in low- and middle-income countries (2, 4). Despite its high prevalence, 70%−80% of COPD cases remain undiagnosed due to the challenges in early detection (5–7).

### Machine learning in disease prediction

Machine learning (ML) has emerged as a transformative tool for COPD screening and risk assessment by analyzing complex, multi-dimensional healthcare data (8–10). For instance, Lin et al. (11) developed a machine learning-based decision system using gradient boosting classifiers (CatBoost, LightGBM, and XGBoost), which achieved an area under the curve (AUC) of 99.85% in identifying high-risk COPD groups. Similarly, Wang et al. (12) created a COPD risk screening model using logistic regression and generalized additive models, with an AUC exceeding 0.8, showing strong predictive performance. Zeng et al. (13) developed a ML model using data from over 43,000 COPD patients, achieving an AUC of 0.866 for predicting severe exacerbations within 1 year, outperforming previous models. These studies highlight the potential of ML to improve COPD screening, enhance diagnostic accuracy, and support more effective interventions.

### Environmental exposures and respiratory health

Global environmental pollution exposure is widespread, with 91% of the world's population living in areas exceeding WHO safety guidelines for pollutants like PM2.5 and ozone (14, 15). Environmental conditions are linked to 24% of all deaths globally, with air pollution alone causing 400,000 premature deaths annually in Europe and reducing average life expectancy by 1 year (16, 17). Niu et al. (18) found that particulate matter exposure increased COPD exacerbation risk, particularly in younger and severe COPD patients. Yan et al. (19) demonstrated that higher blood cadmium and lead levels were associated with increased COPD risk, while anthocyanidin intake above 11.56 mg/day reduced cadmium-related COPD risk by 27%. Madani et al. (20) showed that volatile organic compounds from local sources significantly increased respiratory disease-related emergency room visits, with ethylbenzene having the greatest impact on asthma and COPD. Environmental pollutants pose significant respiratory health risks globally, with effects varying by pollutant type and population vulnerability.

### PFAS exposure: background and health impacts

Per- and polyfluoroalkyl substances (PFAS) are a group of synthetic chemicals widely used in industrial and consumer products due to their exceptional chemical stability, water resistance, and heat resistance (21–23). However, their persistence in the environment and bioaccumulation in human tissues have raised significant public health concerns (24–26). PFAS exposure has been linked to various adverse health outcomes, including metabolic disorders (27), liver damage (28), immune dysfunction (29), and respiratory diseases, such as asthma (30) and reduced lung function (31, 32). Recent studies have also explored the relationship between PFAS and COPD. For instance, Wang et al. analyzed data from the National Health and Nutrition Examination Survey (NHANES) 2007–2018 and found that perfluorooctanoic acid (PFOA) and PFNA exposure significantly increased COPD risk, particularly in males, with a J-shaped dose-response relationship (33, 34). Their study further identified serum albumin as a mediator in the association between PFOA and COPD, with a mediation proportion of 17.94%, suggesting potential pathways involving oxidative stress and chronic inflammation (34). Despite these advancements, research on PFAS and COPD remains limited, and limited studies have applied ML approaches to investigate this relationship or develop predictive models.

### Rationale for model interpretability in public health

Despite emerging evidence linking PFAS exposure to COPD, current research remains limited in both scope and methodology (33, 34). Most existing studies rely on conventional statistical models, which may not fully capture the complex, non-linear relationships between PFAS and COPD risk, nor do they provide individualized risk estimation (35, 36). Moreover, few have explored the use of machine learning to enhance predictive performance or model interpretability in this domain. To address these gaps, our study aims to systematically evaluate the relationship between PFAS exposure and COPD risk using advanced ML approaches. By leveraging nationally representative data from the 2013–2018 NHANES, we developed interpretable ML models to predict individual COPD risk, focusing on performance metrics such as AUC, sensitivity, and specificity. We further applied SHAP and partial dependence analyses to uncover both global and personalized insights into how specific PFAS contribute to COPD risk. Finally, to support real-world application, we translated our findings into an accessible online risk calculator, facilitating early screening and informing prevention strategies in public health practice.

## Method

### Study population

**Figure 1:**
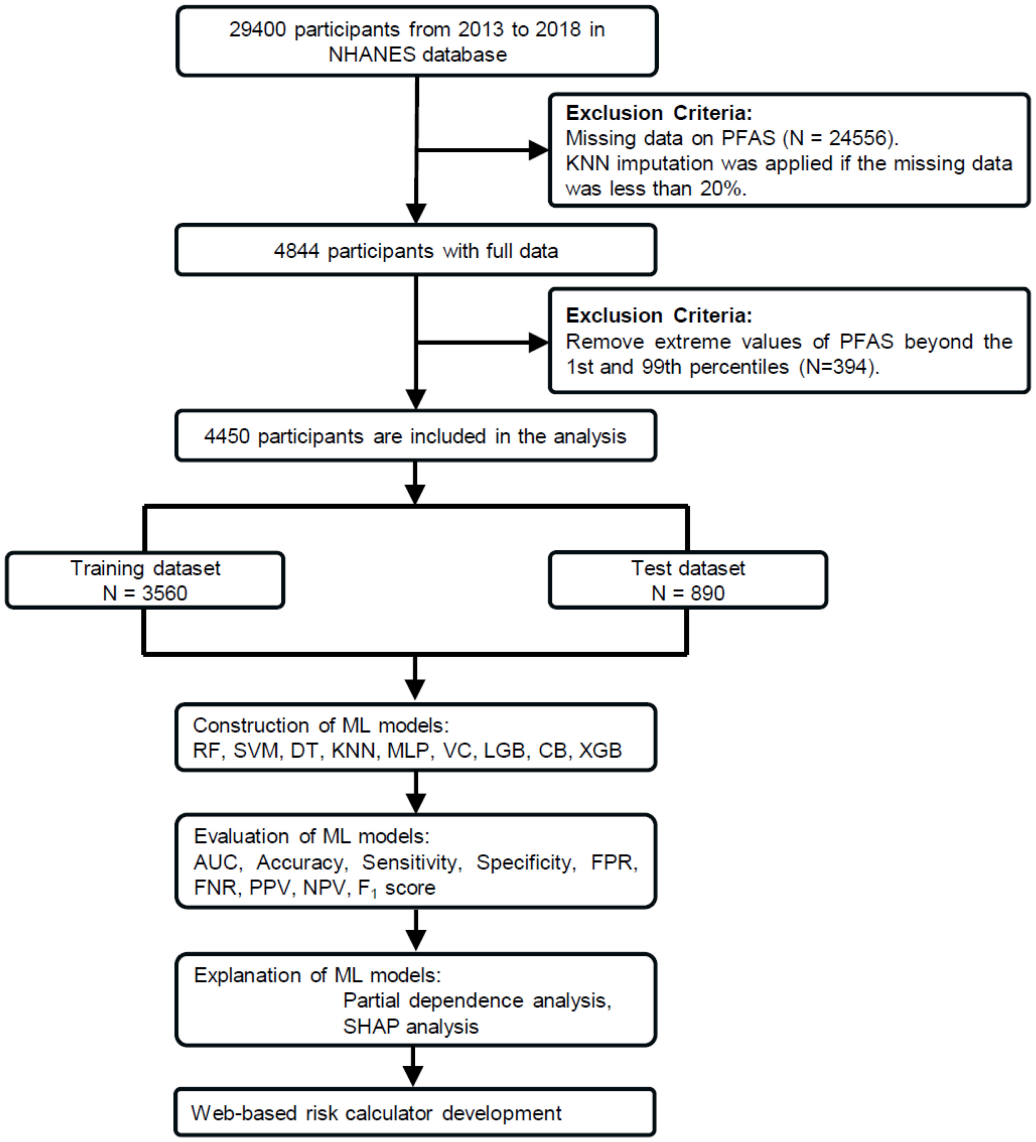
Study workflow for PFAS exposure and COPD risk analysis. From 29,400 NHANES participants (2013–2018), 4,450 were included after data preprocessing. The dataset was split into training (*n* = 3,560) and test (*n* = 890) sets. Nine machine learning (ML) models were trained using these covariates as predictors. The best-performing model (CatBoost) was further analyzed using partial dependence plots (PDP) and SHapley Additive exPlanations (SHAP).

The National Health and Nutrition Examination Survey (NHANES) is a program conducted by the CDC to study the health and nutrition of people living in the United States (34). For this study, we used data from three NHANES cycles (2013–2018), which included 29,400 participants. After excluding individuals with missing covariates or serum PFAS concentration data, 4,844 participants remained. Missing values, present in < 20% of the data, were addressed using the K-nearest neighbors (KNN) imputation method. To ensure robust results, we further excluded extreme PFAS values below the 1st percentile and above the 99th percentile (37), leaving a final sample of 4,450 participants, as shown in Figure 1. All participants provided written informed consent, and the study was approved by the National Center for Health Statistics Research Ethics Review Board.

### Serum PFAS

The PFAS analyzed were 2-(N-Methyl-perfluorooctane sulfonamido) acetic acid (MPAH), perfluorodecanoic acid (PFDE), perfluorohexane sulfonic acid (PFHxS), perfluorononanoic acid (PFNA), perfluorooctane sulfonic acid (PFOS), perfluorooctanoic acid (PFOA), and perfluoroundecanoic acid (PFUA). Total concentrations of PFOS and PFOA were calculated by combining their isomers: linear (n-PFOA) and branched (Sb-PFOA) for PFOA, and linear (n-PFOS) and monomethyl branched (Sm-PFOS) for PFOS. Pearson correlation coefficients were used to evaluate relationships among the seven PFAS.

### Covariates

This study included age, gender, race, education level, marital status, body mass index (BMI), family income, and smoking status as covariates. Race was divided into five categories: Mexican American, other Hispanic, non-Hispanic White, non-Hispanic Black, and other. Education was grouped into two levels: high school or less, and more than high school. Marital status options were married, widowed, divorced, separated, never married, and living with a partner. Family income was calculated as a ratio of family income to poverty guidelines, with any value above 5 recorded as 5. Smoking status was defined as having smoked at least 100 cigarettes over a lifetime. To assess multicollinearity among covariates, we calculated the variance inflation factor (VIF). Variables with a VIF < 10 were retained for model construction, consistent with prior methodological recommendations for avoiding instability in multivariate models (38).

### ML model construction and evaluation

The ML models were built using 15 variables, comprising 10 continuous variables (age, family income, BMI, and seven PFAS biomarkers: MPAH, PFDE, PFHxS, PFNA, PFOA, PFOS, and PFUA) and five categorical variables (gender, race, education level, marital status, and smoking status). Continuous variables were standardized using StandardScaler from scikit-learn to ensure zero mean and unit variance. Categorical variables were encoded as integers without additional transformation. The dataset was randomly split into training (80%, *n* = 3,560) and testing (20%, *n* = 890) sets using stratified sampling to maintain the proportion of COPD cases in both sets.

Nine machine learning algorithms were implemented using Python 3.9.19 and scikit-learn 1.3.0: random forest (RF), support vector machine (SVM), decision tree (DT), K-nearest neighbors (KNN), multilayer perceptron (MLP), voting classifier (VC), light gradient boosting machine (LightGBM), CatBoost, and Extreme Gradient Boosting (XGBoost). These models were chosen based on their demonstrated performance in prior studies involving clinical or environmental health prediction tasks (39, 40).

Hyperparameter tuning was performed using grid search, with the optimized parameters provided in Supplementary Table S1. The workflow of the study is shown in Figure 1. Model performance was evaluated using metrics such as the receiver operating characteristic (ROC) curve, area under the curve (AUC), accuracy, sensitivity (recall), specificity, false-positive rate (FPR), false-negative rate (FNR), positive predictive value (PPV), negative predictive value (NPV), and *F*_1_ score. These metrics are widely used in medical machine learning studies to assess both discriminatory power and classification balance, especially under imbalanced conditions (39, 41).

### ML model interpretation

To analyze the impact of individual PFAS on COPD risk, partial dependence plots (PDPs) were created using the sklearn.inspection module with a grid resolution of 50 points. These plots demonstrate how a specific feature influences the model's predictions while holding other variables constant. Using the trained CatBoost model, the relationship between selected features and COPD risk was calculated and visualized. The trends were smoothed using B-spline interpolation (scipy.interpolate.splrep with smoothing parameter s = 30) to enhance readability, and individual variability was highlighted through sample-specific curves. Additionally, rug plots were included to show the distribution of feature values, providing a deeper understanding of their range within the dataset.

SHapley Additive exPlanations (SHAP) analysis was applied to understand how individual features influenced the predictions made by the trained CatBoost model (42). The SHAP values, calculated using “TreeExplainer,” provided a breakdown of each feature's contribution to the model output. A combined visualization was created, consisting of a dot plot to display the distribution and direction of feature impacts and a bar plot to rank features by their average contribution. This dual representation provided a clear view of the importance and variability of each feature, offering valuable insights into the factors driving COPD risk predictions. All analysis code and data are made publicly available at https://huggingface.co/spaces/MLML202512/COPD/tree/main for reproducibility.

### Web-based risk calculator development

To translate the trained machine learning model into a user-friendly application, we developed an interactive web-based COPD risk calculator using the Gradio framework (https://www.gradio.app/). The calculator was built based on the final CatBoost model, which was trained using selected demographic, socioeconomic, lifestyle, and PFAS biomarker variables. Only the numeric features were standardized using StandardScaler, consistent with the model training pipeline, while categorical variables were kept in their original format as encoded integers. The interface allows users to input raw values for 15 features, including five categorical (gender, race, education level, marital status, and smoking) and 10 numeric variables (age, family income, BMI, and seven PFAS biomarkers: MPAH, PFDE, PFHxS, PFNA, PFOA, PFOS, and PFUA). Upon input, the backend applies the same preprocessing pipeline and uses the trained CatBoost model to generate a binary prediction (COPD or Healthy), a probability score, and a qualitative risk level categorized as low, medium, or high.

### Statistical analysis

Continuous variables were reported as means with standard deviations (SD), and categorical variables as counts with percentages. *T*-tests and chi-square tests were used to compare PFAS levels and demographics between COPD and non-COPD groups. Analyses were performed using Python (3.9.19) and R (4.4.0), with *p*-value < 0.05 considered significant (43).

## Result

### Baseline characteristics

Among 4,450 participants, as shown in Table 1, 180 (4.0%) had COPD. Participants with COPD were older (64.6 ± 11.5 vs. 49.0 ± 17.6 years, *p*-value < 0.001) and more likely to be non-Hispanic White (61.7 vs. 36.5%, *p*-value < 0.001) or have a lower education level (60.0 vs. 43.7%, *p*-value < 0.001). Marital status also differed, with more widowed individuals in the COPD group (15.6% vs. 6.8%, *p*-value < 0.001). While smoking prevalence was lower in the COPD group (13.9 vs. 59.7%, *p*-value < 0.001), this may reflect smoking cessation after diagnosis or survivor bias. PFAS analysis showed higher levels of MPAH (*p*-value < 0.001), lower PFDE (*p*-value = 0.004), and lower PFUA (*p*-value = 0.006) in the COPD group, with no significant differences for PFHxS, PFNA, PFOA, or PFOS.

**Table 1:** Demographic and clinical features of the participants.

**Characteristics**	**Non-COPD (*N* = 4,270)**	**COPD (*N* = 180)**	**Total (*N* = 4,450)**	***p* value**
Age, year	49.0 (17.6)	64.6 (11.5)	49.6 (17.6)	< 0.001
**Gender**, ***n*** **(%)**				
Male	2,012 (47.1%)	96 (53.3%)	2,108 (47.4%)	0.263
Female	2,258 (52.9%)	84 (46.7%)	2,342 (52.6%)	
**Race**, ***n*** **(%)**				
Mexican American	690 (16.2%)	8 (4.4%)	698 (15.7%)	< 0.001
Other Hispanic	481 (11.3%)	15 (8.3%)	496 (11.1%)	
Non-Hispanic White	1,558 (36.5%)	111 (61.7%)	1,669 (37.5%)	
Non-Hispanic Black	909 (21.3%)	26 (14.4%)	935 (21.0%)	
Other	632 (14.8%)	20 (11.1%)	652 (14.7%)	
**Education**, ***n*** **(%)**				
High school or lower	1,864 (43.7%)	108 (60.0%)	1,972 (44.3%)	< 0.001
More than high school	2,406 (56.3%)	72 (40.0%)	2,478 (55.7%)	
**Marital status**, ***n*** **(%)**				
Married	2,191 (51.3%)	79 (43.9%)	2,270 (51.0%)	< 0.001
Windowed	291 (6.8%)	28 (15.6%)	319 (7.2%)	
Divorced	448 (10.5%)	37 (20.6%)	485 (10.9%)	
Separated	158 (3.7%)	5 (2.8%)	163 (3.7%)	
Never married	808 (18.9%)	27 (15.0%)	835 (18.8%)	
Living with partner	374 (8.8%)	4 (2.2%)	378 (8.5%)	
Weight, kg	82.7 (22.1)	86.2 (27.8)	82.8 (22.4)	0.558
Height, cm	167 (10.2)	166 (10.4)	167 (10.2)	0.985
BMI, kg/m^2^	29.7 (7.13)	31.1 (9.73)	29.8 (7.26)	0.480
Family income	2.52 (1.61)	1.80 (1.22)	2.49 (1.60)	< 0.001
Smoke, *n* (%)	2,550 (59.7%)	25 (13.9%)	2,575 (57.9%)	< 0.001
MPAH, ng/ml	0.161 (0.184)	0.231 (0.237)	0.164 (0.187)	< 0.001
PFDE, ng/ml	0.236 (0.202)	0.194 (0.182)	0.234 (0.202)	0.004
PFHxS, ng/ml	1.63 (1.45)	1.72 (1.42)	1.63 (1.44)	0.403
PFNA, ng/ml	0.695 (0.470)	0.636 (0.437)	0.692 (0.469)	0.188
PFOA, ng/ml	1.88 (1.16)	2.00 (1.18)	1.89 (1.16)	0.323
PFOS, ng/ml	6.63 (5.32)	6.75 (5.07)	6.64 (5.31)	0.782
PFUA, ng/ml	0.153 (0.138)	0.120 (0.0970)	0.152 (0.137)	0.006

BMI, body mass index; MPAH, 2-(N-Methyl-perfluorooctane sulfonamido) acetic acid; PFDE, perfluorodecanoic acid; PFHxS, perfluorohexane sulfonic acid; PFNA, perfluorononanoic acid; PFOS, perfluorooctane sulfonic acid; PFOA, perfluorooctanoic acid; PFUA, perfluoroundecanoic acid.

Serum PFAS concentrations showed significant changes from 2013 to 2018 (*p*-value < 0.001), as shown in Table 2. PFHxS, PFNA, PFOA, and PFOS levels declined over time, with PFOS dropping from 6.91 ng/ml in 2013–2014 to 6.22 ng/ml in 2017–2018, and PFOA from 2.23 to 1.62 ng/ml. MPAH, PFDE, and PFUA levels remained relatively stable. These trends suggest reduced PFAS exposure, likely due to regulatory measures and shifts in industrial practices. The Pearson correlation analysis showed strong relationships between PFUA and PFDE (*r* = 0.74) and PFOS with PFNA (*r* = 0.62), while MPAH exhibited weak correlations with other PFAS (Supplementary Figure S1). These results suggest shared sources or pathways for certain PFAS.

**Table 2:** Serum concentration of PFAS from 2013 to 2018.

**PFAS**	**NHANES cycles**				***p* value**
	**2013–2014 (*N* = 1,444)**	**2015–2016 (*N* = 1,506)**	**2017–2018 (*N* = 1,500)**	**Total (*N* = 4,450)**	
MPAH, ng/ml	0.169 (0.194)	0.153 (0.183)	0.171 (0.183)	0.164 (0.187)	< 0.001
PFDE, ng/ml	0.250 (0.210)	0.209 (0.199)	0.245 (0.194)	0.234 (0.202)	< 0.001
PFHxS, ng/ml	1.82 (1.54)	1.62 (1.46)	1.46 (1.31)	1.63 (1.44)	< 0.001
PFNA, ng/ml	0.817 (0.494)	0.730 (0.473)	0.535 (0.390)	0.692 (0.469)	< 0.001
PFOA, ng/ml	2.23 (1.29)	1.82 (1.09)	1.62 (1.01)	1.89 (1.16)	< 0.001
PFOS, ng/ml	6.91 (5.21)	6.79 (5.48)	6.22 (5.21)	6.64 (5.31)	< 0.001
PFUA, ng/ml	0.157 (0.152)	0.133 (0.121)	0.165 (0.135)	0.152 (0.137)	< 0.001

MPAH, 2-(N-Methyl-perfluorooctane sulfonamido) acetic acid; PFAS, Per- and polyfluoroalkyl substances; PFDE, perfluorodecanoic acid; PFHxS, perfluorohexane sulfonic acid; PFNA, perfluorononanoic acid; PFOS, perfluorooctane sulfonic acid; PFOA, perfluorooctanoic acid; PFUA, perfluoroundecanoic acid.

### ML models construction and evaluation

**Figure 2:**
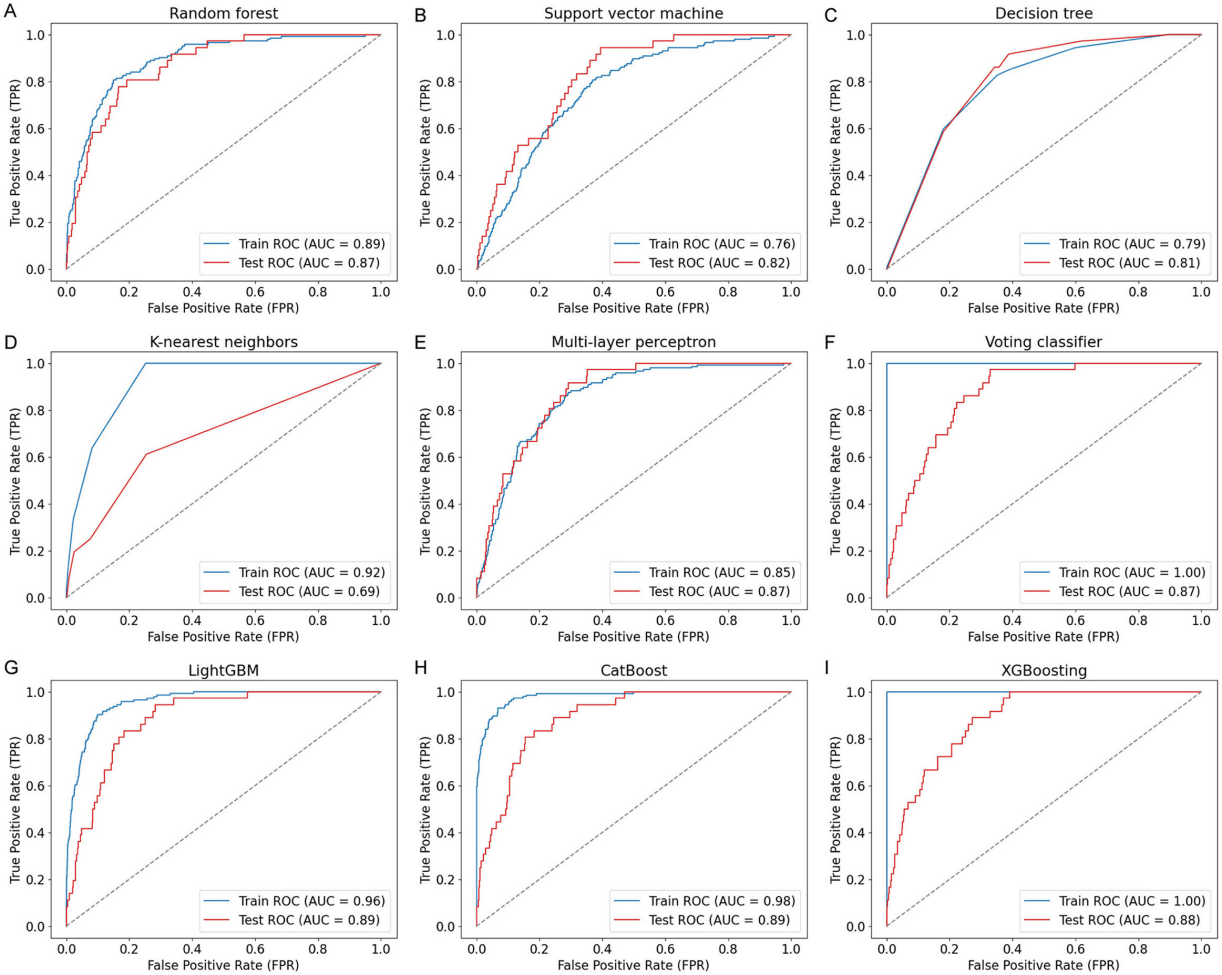
ROC curves of nine ML models for COPD prediction. ROC curves **(A–I)** illustrate the model performance on both training and test sets using covariates including age, sex, BMI, smoking status, family income, and seven PFAS biomarkers. The nine models include: **(A)** Random Forest (RF), **(B)** Support Vector Machine (SVM), **(C)** Decision Tree (DT), **(D)** K-nearest neighbors (KNN), **(E)** Multi-Layer Perceptron (MLP), **(F)** Voting Classifier (VC), **(G)** LightGBM (LGB), **(H)** CatBoost (CB), and **(I)** XGBoost (XGB). CatBoost achieved the highest test AUC of 0.89.

Nine ML models, including RF, SVM, DT, KNN, MLP, VC, LGB, CB, and XGB, were constructed and evaluated to predict COPD risk. Performance metrics such as AUC, accuracy, sensitivity, and specificity were used to assess the models, as shown in Table 3. Among these, CatBoost emerged as the best-performing model, achieving the highest accuracy (84%), AUC (0.89), sensitivity (81%), and specificity (84%). The ROC curves in Figure 2 further confirmed the robust performance of CatBoost, showing minimal overfitting and consistent AUC values between training and testing datasets. In contrast, other models like KNN exhibited significant overfitting, with a large performance gap between training (AUC = 0.92) and testing (AUC = 0.69). Given its superior performance, CatBoost was selected as the final model for further analysis.

**Table 3:** Discrimination characteristics among nine ML models.

**Metrics**	**RF**	**SVM**	**DT**	**KNN**	**MLP**	**VC**	**LGB**	**CB**	**XGB**
AUC	0.87	0.82	0.81	0.69	0.87	0.87	0.89	0.89	0.88
Accuracy (%)	84	60	62	74	71	71	73	84	73
Sensitivity/Recall	0.69	0.94	0.92	0.61	0.92	0.89	0.89	0.81	0.89
Specificity	0.85	0.59	0.61	0.75	0.70	0.70	0.73	0.84	0.73
FPR	0.15	0.41	0.39	0.25	0.30	0.30	0.27	0.16	0.27
FNR	0.31	0.06	0.08	0.39	0.08	0.11	0.11	0.19	0.11
PPV	0.16	0.09	0.09	0.09	0.11	0.11	0.12	0.18	0.12
NPV	0.99	1.00	0.99	0.98	0.99	0.99	0.99	0.99	0.99
*F*_1_ score	0.26	0.16	0.17	0.16	0.20	0.20	0.21	0.29	0.21

RF, random forest; SVM, support vector machine; DT, decision tree; KNN, K-nearest neighbors; MLP, multilayer perceptron; VC, voting classifier; LGB, light gradient boosting machine; CB, CatBoost; XGB, extreme gradient boosting; AUC, area under the receiver operating characteristic curve; Accuracy (%), Percentage of correctly classified samples; Sensitivity/Recall, true positive rate; Specificity, true negative rate; FPR, false positive rate; FNR, false negative rate; PPV, positive predictive value; NPV, negative predictive value; F_1_ score, Harmonic mean of precision and recall.

### ML models interpretation

**Figure 3:**
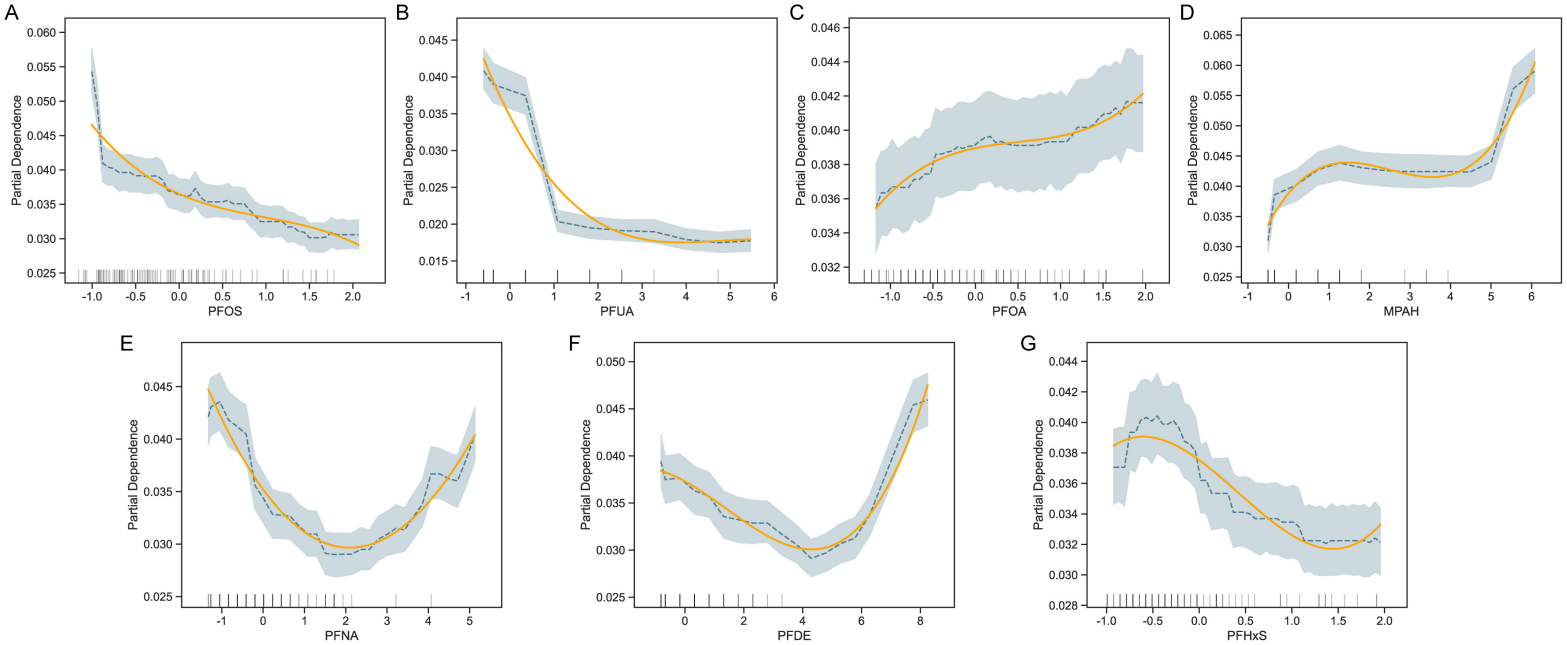
Partial Dependence Plots (PDP) for PFAS and COPD risk. PDPs for selected PFAS predictors—PFOS, PFUA, PFOA, MPAH, PFNA, PFDE, and PFHxS **(A–G)**—illustrate the marginal effect of each feature on predicted COPD risk, while holding other covariates constant. For each panel, shaded bands indicate 95% confidence intervals and rug plots show the distribution of data points. Adjusted covariates include demographic and behavioral variables such as age, sex, BMI, smoking status, and income level.

To investigate the relationship between specific PFAS exposure and COPD risk, we performed partial dependence analysis in the trained CatBoost model (Figure 3). The results revealed varying, non-linear associations for different PFAS. COPD risk decreased with higher levels of PFOS and PFUA, suggesting a potential protective effect, while PFOA and MPAH showed a positive association, with risk increasing at higher concentrations. PFNA exhibited a U-shaped relationship, indicating increased risk at both low and high levels, while moderate levels were associated with lower risk. PFDE demonstrated a decreasing trend in risk at moderate levels, followed by an increase at higher concentrations. PFHxS showed a fluctuating pattern without a clear monotonic trend. These findings highlighted the complex influence of PFAS on COPD risk, suggesting that different PFAS may affect the disease through distinct mechanisms.

**Figure 4:**
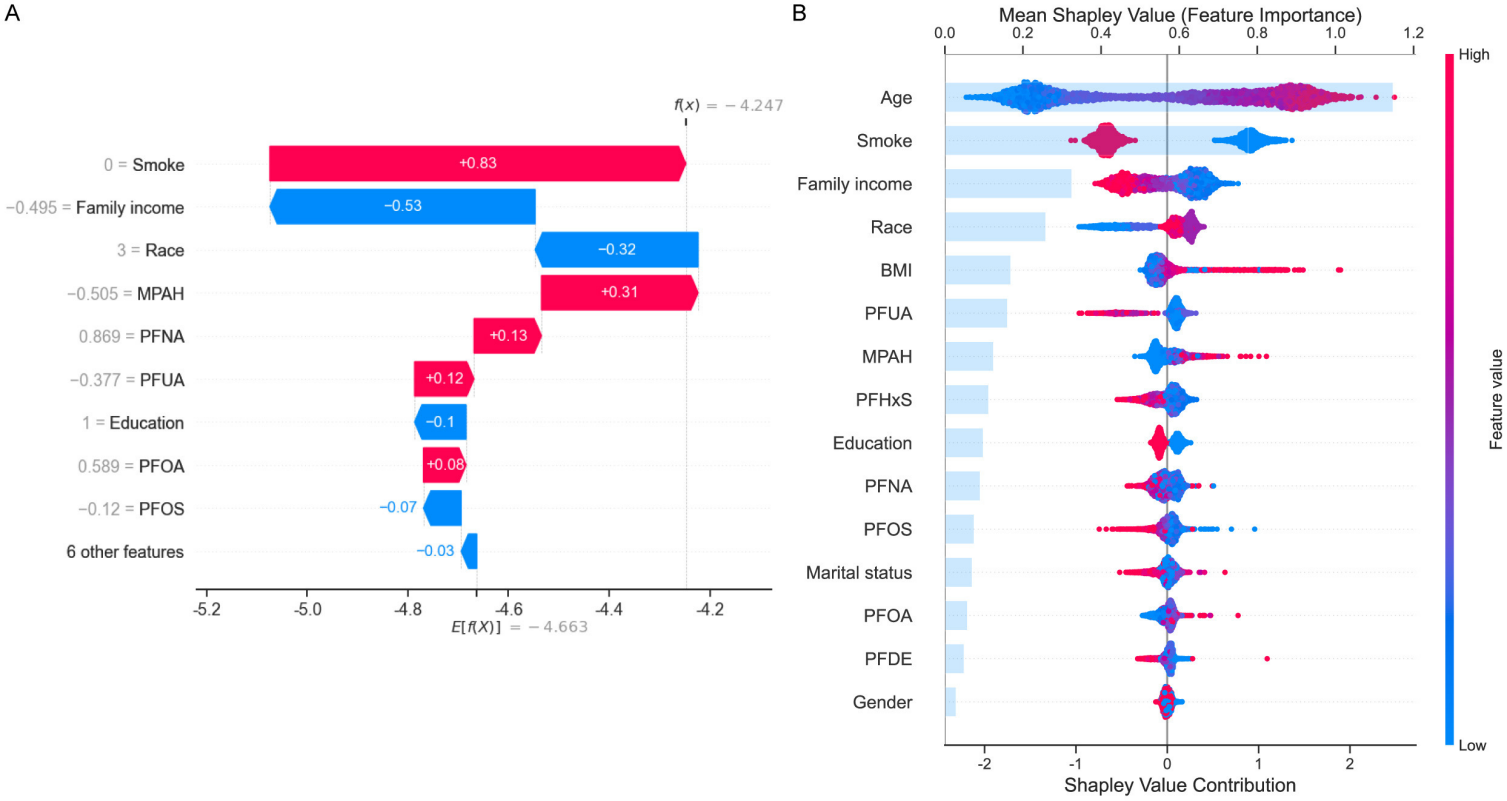
SHapley Additive exPlanations (SHAP) analysis for COPD risk prediction. **(A)** Waterfall plot showing the contribution of top features (e.g., smoke, family income, MPAH, PFNA, PFUA) to an individual prediction. Positive (red) values increase risk, while negative (blue) values reduce risk. **(B)** Summary plot displaying mean SHAP values for all features across the dataset, ranked by importance. Age and smoke are the strongest predictors, with PFAS (PFUA, PFOS, PFOA, MPAH, and PFNA) showing varied directional impacts on COPD risk. The color gradient represents feature values, with red indicating high values and blue low values.

To further interpret the contributions of individual features to COPD risk, SHAP analysis was performed. Figure 4A illustrated a waterfall plot, which highlighted the impact of key features on an individual prediction. Smoking status had the largest positive contribution to COPD risk, followed by PFNA and MPAH. Conversely, family income and PFUA were associated with reduced risk. The plot clearly showed how individual features influenced the model's prediction for a specific instance. Figure 4B presented a summary plot of SHAP values across the entire dataset, ranking features by their overall importance. Age was the most significant contributor to COPD risk, with older age associated with higher risk. Among PFAS, PFUA, PFHxS, and PFOS demonstrated negative contributions, indicating that lower levels of these PFAS were linked to higher COPD risk. Conversely, MPAH and PFOA showed positive contributions, meaning that higher levels were associated with increased risk. PFNA and PFDE exhibited a mixed effect, with both low and high levels contributing differently to the risk. The SHAP summary plot illustrated these trends, with red indicating feature values that increase COPD risk and blue indicating values that decrease COPD risk, providing a clear and detailed understanding of the directionality of each PFAS's impact on COPD risk.

### Web-based risk calculator

**Figure 5:**
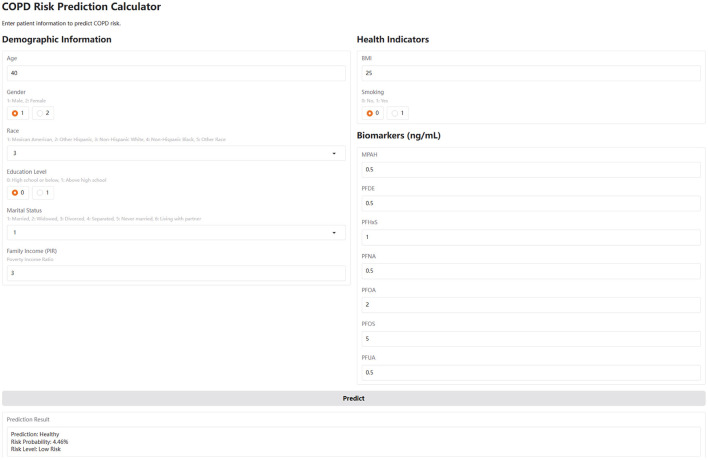
The web-based COPD risk prediction calculator. This calculator, developed using the Gradio framework, integrates the trained CatBoost model. Users input values for age, sex, BMI, smoking, income, and serum PFAS levels. The tool applies the same standardization and feature scaling as in model training, and outputs a COPD risk probability, risk category (Low/Medium/High), and binary prediction (COPD or Healthy). It is accessible at: https://huggingface.co/spaces/MLML202512/COPD.

To enhance accessibility and clinical applicability, we implemented a web-based COPD risk calculator using the Gradio framework. This interactive tool integrates the trained CatBoost model and allows users to input raw demographic, lifestyle, and PFAS biomarker data through a browser interface (Figure 5). The calculator automatically standardizes numeric features in the backend and provides real-time predictions, including binary classification (COPD or Healthy), probability of risk, and a qualitative risk level (low, medium, or high). The web-based calculator serves as a user-friendly prototype for personalized risk assessment and may assist clinicians or public health professionals in early identification and stratification of COPD risk, particularly in PFAS-exposed populations (https://huggingface.co/spaces/MLML202512/COPD).

## Discussion

### Summary of main findings and model performance

This study is the first to use interpretable ML techniques to investigate the association between PFAS exposure and COPD risk, utilizing data from the US NHANES (2013–2018). Among the nine ML models tested, CatBoost emerged as the best performer, achieving an accuracy of 84%, AUC of 0.89, sensitivity of 81%, and specificity of 84%, making it the optimal choice for predicting COPD risk. To provide deeper insights, feature importance analysis, partial dependence plots and SHAP analysis were conducted to evaluate how individual PFAS and other factors influence COPD risk. These findings underscored the importance of regulating PFAS exposure to mitigate health risks and demonstrated the potential of interpretable ML methods to identify high-risk populations, guiding targeted interventions and improving public health outcomes.

### PFAS as key predictors of COPD risk: consistency with prior studies

Previous research highlights that PFOA and PFNA are strongly associated with increased COPD risk, particularly among males, exhibiting a characteristic nonlinear and J-shaped dose-response relationship for PFOA exposure (34). Similarly, Pan et al. demonstrated significant associations between serum levels of PFOS and PFOA and increased COPD risk, noting differential impacts based on sex, age, and smoking status, and indicating protective roles of moderate-intensity physical activity in mitigating PFAS-related COPD risk (33). Our study aligned with these findings, as the CatBoost model identified PFAS, particularly MPAH and PFOA, as significant predictors of COPD risk. Notably, our study uniquely identified PFOS and PFUA as potentially protective against COPD risk, differing from findings reported by Wang et al. (34) and Pan et al. (33), who found positive associations for PFOS. These discrepancies may result from variations in demographic characteristics, exposure measurement methodologies, or different adjustments for confounding variables across studies. While previous literature suggests that PFAS may influence COPD development through inflammation and oxidative stress pathways (44, 45), the specific biological roles of individual PFAS compounds like PFOS and PFUA remain complex and heterogeneous. Thus, further longitudinal and mechanistic studies are needed to clarify these differences and establish causality. Moreover, SHAP analysis in our study highlighted the notable contribution of PFAS to COPD risk, alongside demographic and socioeconomic factors. These results reinforced the hypothesis that PFAS may influence COPD development through mechanisms such as inflammation and oxidative stress (44, 45), further emphasizing the need for stricter PFAS regulation and further exploration of their impact on respiratory health.

### Biological mechanisms underlying PFAS–COPD associations

The observed relationships between PFAS levels and COPD risk in our study can be explained by underlying biological mechanisms, including inflammation (44), oxidative stress (45), and PFAS interactions with albumin and lung tissues (46). For PFOS and PFUA, the protective association at higher concentrations may reflect their ability to stabilize pulmonary surfactants and reduce oxidative stress (47). Albumin, known to bind PFOS and PFUA, could facilitate their targeted delivery to lung tissues (46), while moderate and lower levels might help maintain epithelial integrity (48) and mitigate inflammation (49), key drivers of COPD progression. In contrast, PFOA and MPAH were positively associated with COPD risk at higher concentrations, which aligned with their known pro-inflammatory and oxidative effects (50). PFOA has been shown to activate the NLRP3 inflammasome and increase cytokine production, including IL-6 and TNF-α, leading to sustained inflammation in lung tissues (51). MPAH may exert similar effects by disrupting epithelial barriers and exacerbating oxidative stress (52), contributing to airway damage and disease progression. These findings highlighted the role of chronic inflammation and oxidative damage as central mechanisms linking higher PFOA and MPAH levels to increased COPD risk.

### Non-linear effects of PFNA, PFDE, and PFHxS

The U-shaped relationship observed with PFNA and the mixed pattern with PFDE reflected their dual roles in COPD risk. At moderate concentrations, PFNA and PFDE may exhibit stabilizing effects on lung tissues, potentially reducing inflammation and oxidative stress. However, at very low or high concentrations, these PFAS may disrupt immune homeostasis and amplify inflammatory responses, leading to increased COPD risk (34). The fluctuating trend for PFHxS likely stems from its complex interplay with inflammatory and antioxidant pathways, which may vary depending on individual susceptibility and exposure levels (34). These findings emphasized the nuanced and concentration-dependent effects of PFAS on COPD risk, highlighting the importance of further mechanistic studies to better understand their roles in respiratory health. These findings emphasized the need for further toxicological studies to elucidate the specific mechanisms by which different PFAS contribute to COPD risk. Experimental research is also needed to determine whether certain PFAS exhibit synergistic or antagonistic effects, particularly in cases of mixed exposure. Understanding these interactions will be critical for developing targeted strategies to mitigate the health impacts of PFAS exposure and for informing regulatory policies aimed at reducing risks associated with these persistent environmental pollutants.

### Study limitations

This study has several limitations. First, as NHANES used a multi-stage stratified sampling design, the findings may not fully represent the entire U.S. population. Second, while our machine learning models demonstrated strong predictive performance, they lack external validation on independent datasets, which is essential to assess model stability and generalizability. Third, COPD status in NHANES was based on self-reported questionnaire data rather than spirometry or clinical diagnosis, which may lead to recall bias or disease misclassification. Additionally, smoking status was also self-reported and may be subject to underreporting, particularly among certain demographic groups. Fourth, although we adjusted for several known covariates, potential unmeasured confounders such as physical activity, dietary factors, occupational exposures, and access to healthcare services were not available in our dataset. These variables could influence both PFAS exposure and COPD risk and may have biased the observed associations. Fifth, PFAS concentrations were measured at a single time point, which may not accurately reflect long-term or cumulative exposure levels. Given the chronic nature of COPD, longer-term exposure assessments would provide a more accurate understanding of causal relationships. Furthermore, the exclusion of participants with missing data may have introduced sampling bias, and the lack of access to detailed healthcare records—such as medication history, comorbidities, or imaging findings—limited our ability to fully characterize disease severity or differentiate COPD subtypes. Moreover, this study did not formally compare models with and without PFAS variables, which may limit the assessment of their specific contribution to COPD risk prediction. Finally, cultural and regional differences in environmental exposure, healthcare access, and disease awareness may limit the generalizability of these findings to other populations or countries. These limitations underscore the need for further longitudinal studies incorporating detailed clinical records, long-term exposure measurements, and more comprehensive confounding adjustment to validate and expand upon our findings.

## Conclusion

This study explored the relationship between PFAS exposure and COPD risk using NHANES (2013–2018) data, applying interpretable machine learning techniques for the first time. Among the nine models, CatBoost performed best, achieving an accuracy of 84%, an AUC of 0.89, a sensitivity of 81%, and a specificity of 84%, making it the optimal model. PDP analysis revealed that higher PFOS and PFUA levels were associated with reduced COPD risk, while higher PFOA and MPAH increased risk. PFNA, PFHxS, and PFDE showed complex, non-linear associations. SHAP analysis provided individual risk predictions and overall variable contributions, while an interactive web-based calculator was deployed for real-time risk assessment. This is the first study to integrate interpretable ML algorithms with large-scale epidemiological data to examine concentration-dependent effects of individual PFAS compounds on COPD risk. By combining advanced modeling with user-friendly tools, our approach bridges data science and clinical application. These results emphasize the need for PFAS regulatory actions and demonstrate how transparent ML can enhance precision risk stratification in chronic respiratory diseases, providing a scalable framework adaptable to other environmental exposures and health outcomes.

## Data Availability

The original contributions presented in the study are included in the article/Supplementary material, further inquiries can be directed to the corresponding authors.
