# Supplementary figures and images for: Developing an interpretable machine learning predictive model of chronic obstructive pulmonary disease by serum PFAS concentration
Source: Front Public Health. 2025 Jul 10;13:1602566. doi: 10.3389/fpubh.2025.1602566 (PMC12287074; doi:10.3389/fpubh.2025.1602566)

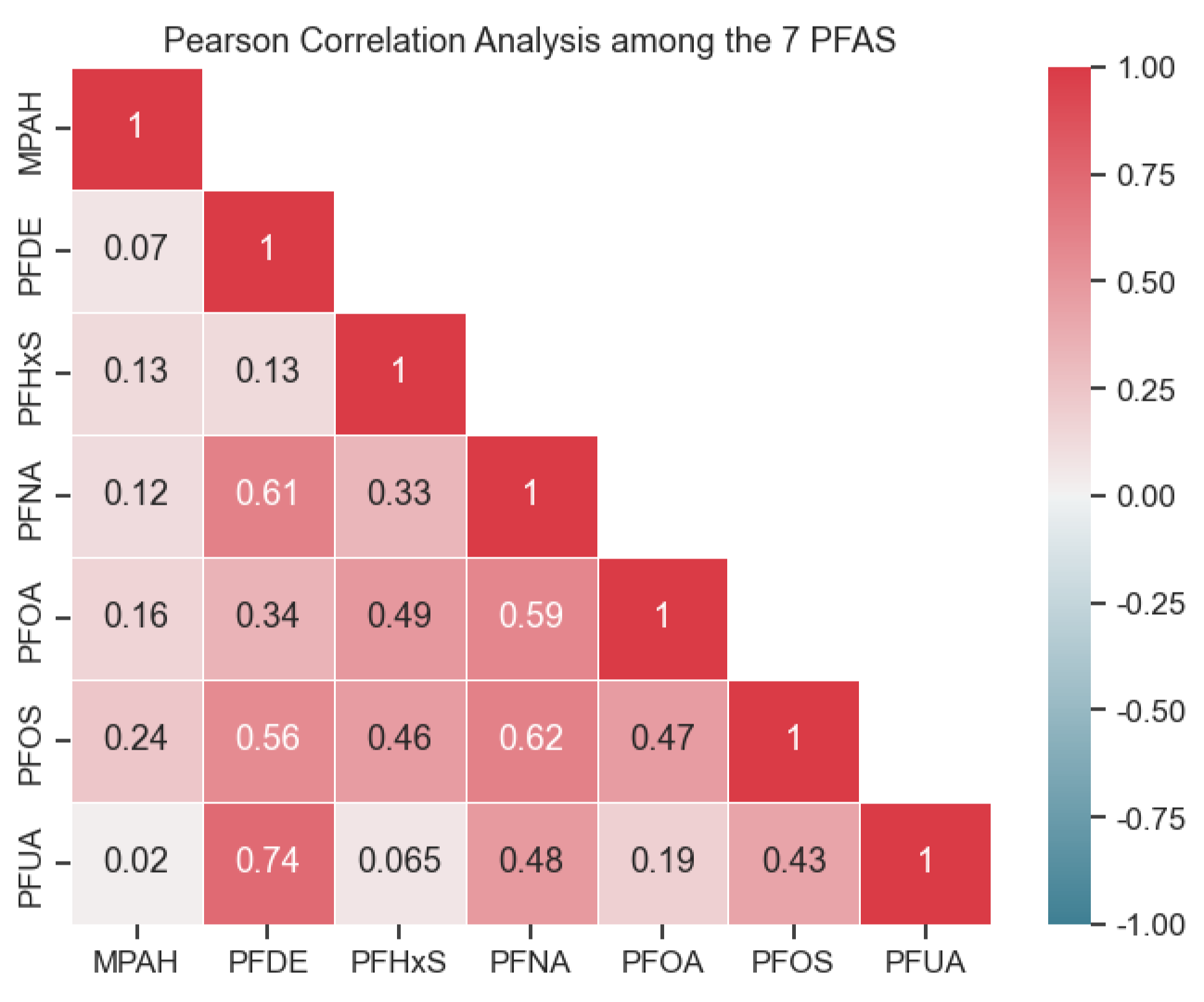

Supplement: Supplementary file 2 [file Image_1.tif]
